# Development of gene expression system in egg cells and zygotes isolated from rice and maize

**DOI:** 10.1002/pld3.10

**Published:** 2017-09-06

**Authors:** Narumi Koiso, Erika Toda, Masako Ichikawa, Norio Kato, Takashi Okamoto

**Affiliations:** ^1^ Department of Biological Sciences Tokyo Metropolitan University Hachioji Tokyo Japan; ^2^ Plant Breeding Innovation Laboratory RIKEN Innovation Center Tsurumi Yokohama Japan; ^3^ Plant Innovation Center Japan Tobacco Inc. Iwata Shizuoka Japan

**Keywords:** egg cell, gene function, *Oryza sativa*, polyethylene glycol, protoplast, transfection, zea maize, zygote

## Abstract

Polyethylene glycol calcium (PEG‐Ca^2+^) transfection‐mediated analysis allows rapid and efficient examination of gene function. To investigate the diverse cellular functions of genes of interest in plant cells, macromolecules, such as DNA, RNA, and proteins, are delivered into protoplasts prepared from somatic tissues or calli using a PEG‐Ca^2+^ transfection procedure. To take advantage of this macromolecule delivery system in the reproductive and developmental biology of angiosperms, this study established a PEG‐Ca^2+^ transfection system with isolated egg cells and zygotes. The conditions for PEG and plasmid DNA concentrations for transfection of rice egg cells were first addressed, and ~30% of PEG‐Ca^2+^‐transfected egg cells showed exogenous and transient expressions of fluorescent proteins from plasmid DNA delivered into the cells. Interestingly, a dual expression of two different fluorescent proteins in the same egg cell using two kinds of plasmid DNAs was also observed. For PEG‐Ca^2+^ transfection with maize zygotes, ~80% of zygotes showed expression of GFP proteins from plasmid DNA. Importantly, PEG‐transfected zygotes developed normally into cell masses and mature plants. These results suggest that the present PEG‐Ca^2+^‐mediated transient expression system provides a novel and effective platform for expressing and analyzing genes of interest in egg cells and zygotes. Moreover, combined with the CRISPR/Cas9 approach, the present transient expression system in zygotes will become a powerful and alternative tool for the preparation of gene‐edited plants.

**SIGNIFICANCE STATEMENT**

PEG‐Ca^2+^‐mediated plasmid DNA transfection of isolated rice egg cells and maize zygotes resulted in transient expression of target genes, and PEG‐transfected zygotes developed normally into plantlets.

## 1 INTRODUCTION

There are several ways to introduce macromolecules, such as DNA, RNA, and proteins, into plant cells (Davey, Rech, & Mulligan, 1989). In many cases, protoplasts are used as the starting materials because cell walls block access of macromolecules to the plasma membrane, and the removal of cell walls from plant cells provides opportunity for the movement of the macromolecules across the plasma membrane (Antonelli & Stadler, 1989; Armstrong, Petersen, Buchholz, Bowen, & Sulc, 1990; Crossway et al., 1986; Davey, Cocking, Freeman, Pearce, & Tudor, 1980; Krens, Molendijk, Wullems, & Schilperoort, 1982; Lurquin, 1979; Shillito, Saul, Paszkowski, Muller, & Potrykus, 1985). Delivery of DNA into protoplasts isolated from sterile tobacco shoots treated with polyethylene glycol (PEG) together with calcium was reported by Krens et al. (1982), and the PEG‐Ca^2+^ transfection of DNA to protoplasts has been used to study diverse cellular functions of genes of interest (Zhang, McElroy, & Wu, 1991; Sheen, 2001; Yoo, Cho, & Sheen, 2007). In addition to DNA, An, Sawada, Kawaguchi, Fukusaki, and Kobayashi (2005) and Zhai, Sooksa‐nguan, and Vatamaniuk (2009) reported that PEG‐Ca^2+^ delivery of double‐stranded RNA (dsRNA) into protoplasts from *Arabidopsis* seedlings reduced the expression level of the target genes and that the PEG‐Ca^2+^ transfection‐mediated RNA interference approach using protoplasts allows rapid and efficient analyses to investigate the functions of target genes.

Fertilization and subsequent events in angiosperms, such as embryogenesis and endosperm development, occur in the embryo sac deeply embedded in ovular tissue (Guignard, 1899; Nawaschin, 1898; Raghavan, 2003; Russell, 1992). Investigations into the molecular mechanisms of fertilization and early embryogenesis have been impeded by the difficulties in directly researching the biology of the embedded female gametophytes, zygotes, and early embryos (Kranz, 1999; Okamoto, 2011; Wang, Kuang, Russell, & Tian, 2006). In the last decade, gametes, zygotes, and/or early embryos have been successfully used for the identification of genes that are specifically or preferentially expressed in female gametes, zygotes, or early embryos, as it has been hypothesized that these types of genes function in reproductive or developmental processes, such as gamete differentiation, gamete fusion, and early zygotic development (Abiko, Maeda, Tamura, Hara‐Nishimura, & Okamoto, 2013; Anderson et al., 2013; Ning et al., 2006; Ohnishi et al., 2011; Sprunck, Baumann, Edwards, Langridge, & Dresselhaus, 2005; Steffen, Kang, Macfarlane, & Drews, 2007; Wang et al., 2010; Wuest et al., 2010; Yang, Kaur, Kiriakopolos, & McCormick, 2006).

Among the genes enriched in egg cells, *Egg Cell 1* (*EC1*) encoding small cysteine‐rich protein is likely to be involved in the attachment between male and female gametes in fertilized embryo sac (Sprunck et al., 2012). However, the functions of most genes expressing in a gamete‐specific or fertilization‐induced/suppressed manner have not been analyzed. This is most likely because the experimental approach for addressing their molecular functions in gametes and zygotes usually consists of making several transgenic plants and subsequent observation of embryo sacs deeply embedded in ovaries. In the study, to establish an efficient experimental platform for investigating gene functions in gametes and zygotes, we combined the isolation procedures of female gametes and zygotes from rice and maize flowers (Kranz, Bautor, & Lörz, 1991; Uchiumi, Komatsu, Koshiba, & Okamoto, 2006) with the PEG‐Ca^2+^‐mediated DNA‐delivery approach (Yoo et al., 2007; Zhai et al., 2009), which resulted in the transient expression of introduced DNA in egg cells and zygotes. Moreover, zygotes showing transient expression developed into embryo‐like structures and cell masses, and the cell masses further regenerated into plantlets. The PEG‐Ca^2+^‐mediated transient expression system with egg cells/zygotes will provide efficient and effective opportunities for addressing a variety of gametic and zygotic events. In addition, this transient expression system in zygotes can be employed for gene editing procedures using the CRISPR/Cas9 system (Belhaj, Chaparro‐Garcia, Kamoun, & Nekrasov, 2013; Jinek et al., 2012; Mikami, Toki, & Endo, 2015).

## 2 RESULTS AND DISCUSSION

### 2.1 Conditional optimization for PEG‐Ca^2+^ transfection of rice egg cells with plasmid DNA

For monitoring expression derived from plasmid DNA which was delivered into rice egg cells by PEG‐Ca^2+^ transfection, we primarily used the pGFP‐ER, in which DNA sequence encoding GFP protein tagged with signal peptide (SP) and carboxy terminal His‐Asp‐Glu‐Leu (HDEL) sequence was located under the 35S promoter and HSP70 intron, as C‐terminal HDEL tetrapeptide sequence constitutes an endoplasmic reticulum (ER) retention signal (Munro & Pelham, 1987; Pelham, 1989) and the SP‐GFP‐HDEL proteins targeted to the ER are effectively detected in plant cells (Haseloff, Siemering, Prasher, & Hodge, 1997; Hayashi et al., 2001; Ridge, Uozumi, Plazinski, Hurley, & Williamson, 1999). In addition, the 35S promoter with HSP70 intron is known to function as a constitutive and strong promoter in cells of monocot plants (Pang et al., 1996).

**Figure 1:**
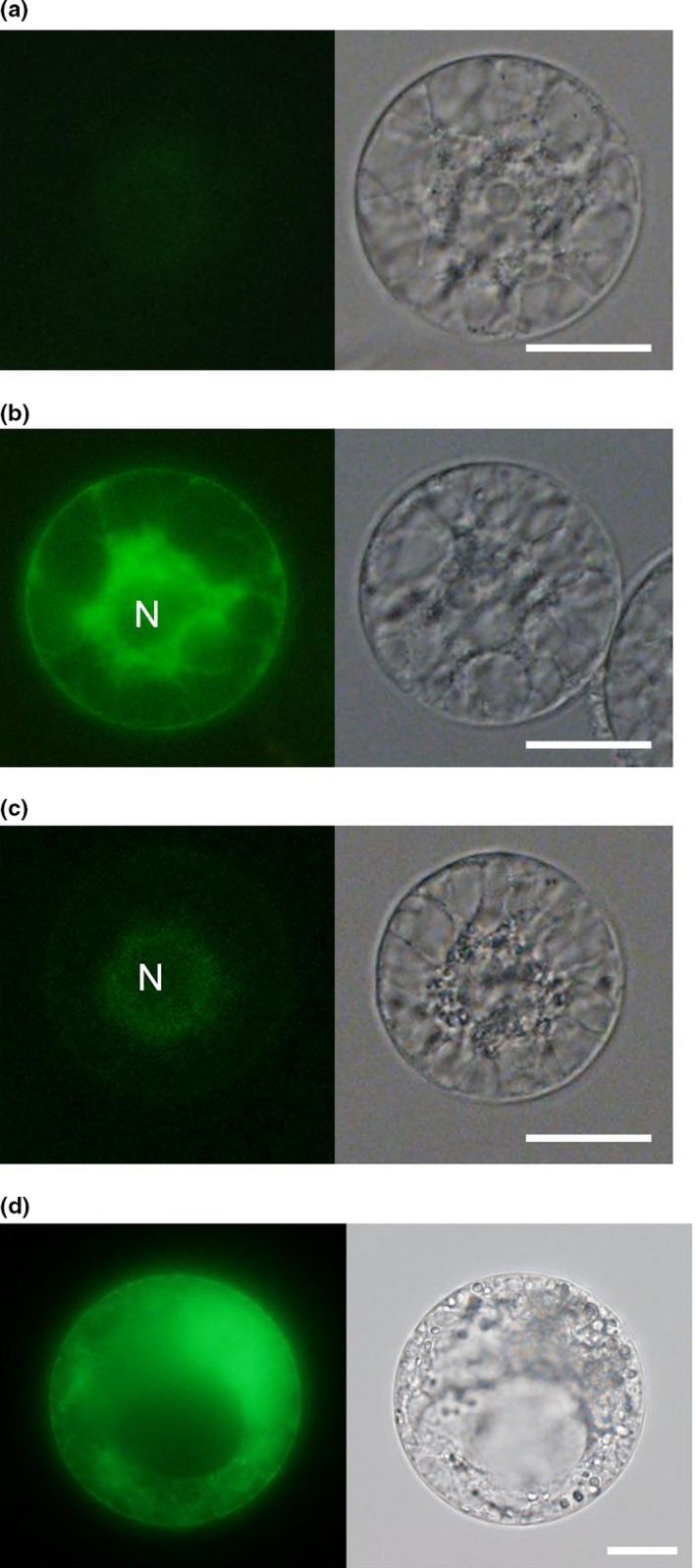
Effect of PEG concentration on expression level of SP‐GFP‐HDEL in rice egg cell (a–c) and maize egg cell (d). (a–c), Rice egg cells were placed in a droplet of MMG solution containing pGFP‐ER on a coverslip, and a droplet of the same volume of PEG solution containing 20% (a) 30% (b) or 40% (c) PEG and 100 mM CaCl_2_ was merged with the MMG droplet holding the egg cell. After washing and 19‐hr culture of the cells, fluorescent signals in egg cells were observed. After washing and 19‐hr culture of the cells, fluorescent signals in egg cells were observed. (d), Maize egg cells were placed in a droplet of MMG solution containing pGFP‐ER on a coverslip, and a droplet of the same volume of PEG solution containing 30% PEG and 100 mM CaCl_2_ was merged with the MMG droplet holding the egg cell. Left and right panels indicate fluorescence and bright‐field images, respectively. N in (b) and (c) indicates egg nucleus. Bars = 20 μm

For successful PEG‐Ca^2+^ transfection using rice egg cells, the concentration of PEG was first addressed. Four egg cells were placed in a droplet of MMG solution (4 mM MES‐KOH pH 5.7, 15 mM MgCl_2_ in mannitol solution of 450 mOsmol/kg H_2_O) containing pGFP‐ER at a concentration of 68 ng/μl, and then, the same volume of a droplet of PEG solution (20% PEG and 100 mM CaCl_2_ in mannitol solution of 450 mOsmol/kg H_2_O) was merged with the MMG droplet where egg cells were held. For other treatments, the same procedures were used except that the PEG solution droplet contained 30% or 40% PEG. After washing and a 19‐hr culture of the treated egg cells, fluorescent signals in egg cells were observed and compared between different PEG concentrations. When using droplets of 20% PEG, no fluorescent signal was detected in any of the four egg cells (Figure 1a; Table S1). When a droplet of 30% PEG was employed, two of the four treated cells showed strong fluorescence derived from SP‐GFP‐HDEL proteins (Figure 1b). Among the four egg cells transfected with a droplet of 40% PEG, only one cell showed a weak SP‐GFP‐HDEL‐derived signal (Figure 1c). Therefore, 30% PEG solution (~15% PEG after the merge of two droplets) was judged as suitable for transfection of egg cells with plasmid DNA. The successful expression in rice egg cells using PEG‐Ca^2+^ transfection is consistent with the report that egg cells isolated from rice flowers are mostly necked cells like protoplasts (Uchiumi et al., 2006), and plasmid DNA appeared to pass through the plasma membrane of the egg cell via well‐conditioned PEG‐Ca^2+^ transfection.

Next, the effect of plasmid DNA concentration on the level of expression of SP‐GFP‐HDEL was examined. Egg cells were placed in MMG droplets containing plasmid DNA at the concentrations of 17, 68, or 272 ng/μl, and PEG‐Ca^2+^ transfection was conducted. For the egg cells located in the 272 ng/μl plasmid droplets, a strong GFP‐derived signal was detected in two of the five treated cells at 13 hr after PEG‐Ca^2+^ transfection, and the fluorescent profile in these five cells did not change at 19 hr after transfection (Table S2). When eight egg cells were PEG‐Ca^2+^‐transfected using an MMG droplet containing 68 ng/μl plasmid DNA, one and three egg cells showed strong and weak fluorescent signals, respectively, at 13 hr after PEG‐Ca^2+^ transfection. Interestingly, at 19 hr of PEG‐Ca^2+^ transfection, a strong GFP signal became detectable in three egg cells in which weak GFP signal was detected at 13 hr (Table S2). In the case of 17 ng/μl plasmid DNA, two of the five treated egg cells showed weak fluorescent signals at 13 hr after PEG‐Ca^2+^ transfection, and the signal in these cells become stronger at 19 hr after PEG‐Ca^2+^ transfection. These results suggest that transient expression of SP‐GFP‐HDEL by PEG‐Ca^2+^ transfection of egg cells with plasmids will occur in a plasmid DNA concentration‐dependent manner in the range of 17–272 ng/μl plasmid DNA. Therefore, we employed a plasmid concentration of 68 or 272 ng/μl in further analyses.

Using the above‐mentioned conditions, maize egg cells were also transfected. Of the nine transfected egg cells, two cells showed SP‐GFP‐HDEL‐derived signal (Fig. 1d and Table 1).

**Table 1:** Efficiency of PEG‐Ca^2+^ transfection of egg cells and zygotes with plasmid DNAs

Cell (plant)	Plasmid	No. of transfected cells	Fluorescent signal		Efficiency (%)
			+	−	
Egg cell (rice)	pGFP‐ER	17	6	11	35.3
	pDsRed	18	6	12	33.3
	pH2B‐GFP	26	5	21	19.2
Egg cell (maize)	pGFP‐ER	9	2	7	22.2
Zygote (rice)	pGFP‐ER	23	0	23	0
Zygote (maize)	pGFP‐ER	19	15	4	78.9

### 2.2 Intracellular localization of exogenously expressed proteins and expression of multiple plasmid DNAs in rice egg cells by PEG‐Ca^2+^ transfection

**Figure 2:**
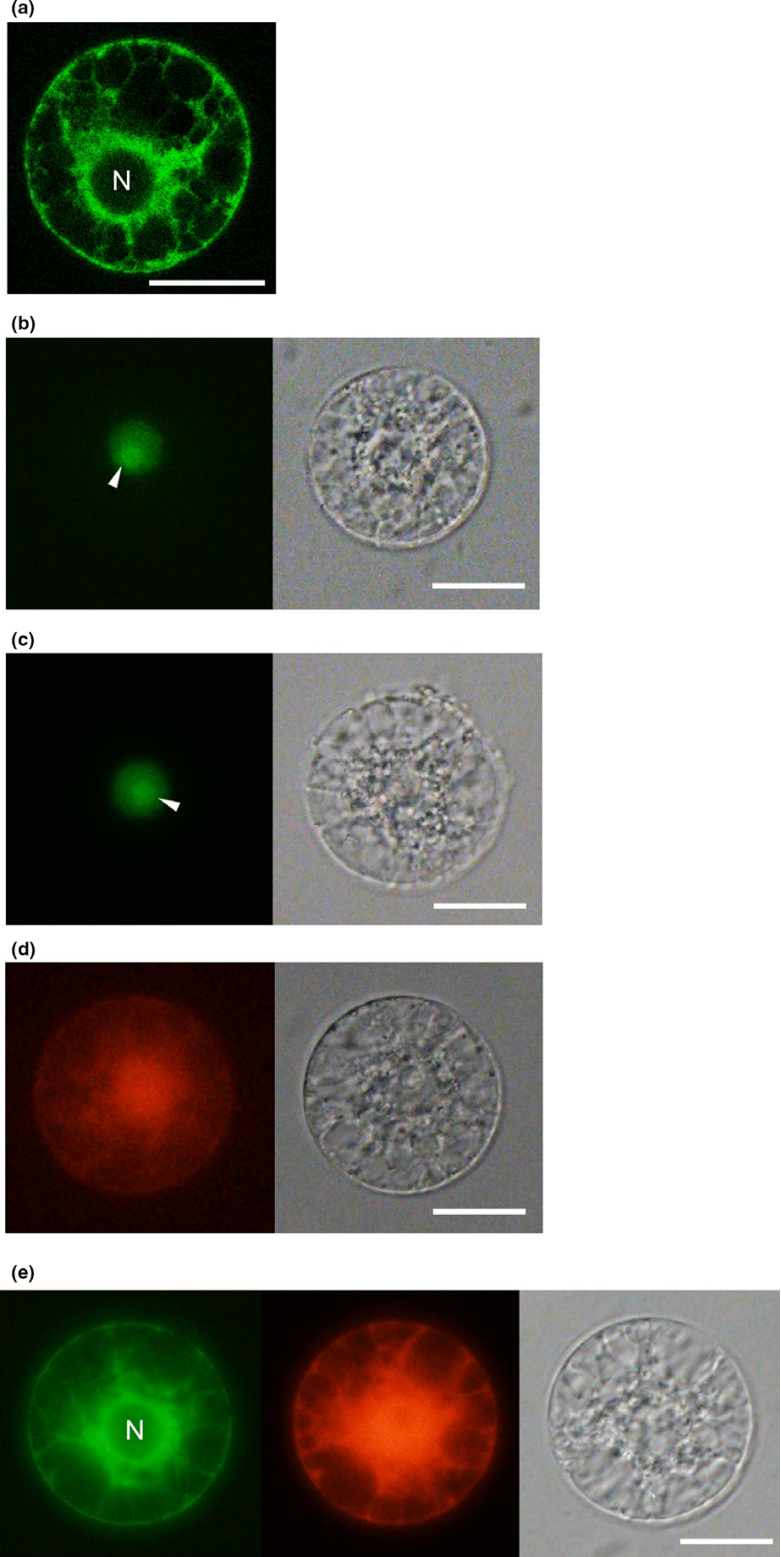
Transient expression of fluorescent proteins in rice egg cells by PEG‐Ca^2+^ transfection with plasmid DNAs. (a), Rice egg cell was PEG‐Ca^2+^‐transfected with pGFP‐ER and observed with a confocal laser scanning microscope. (b), Rice egg cell was PEG‐Ca^2+^‐transfected with pH2B‐GFP and observed with a fluorescent microscope. (c), Egg cell isolated from a flower of a transgenic rice plant expressing H2B‐GFP was observed with a fluorescent microscope. (d), Rice egg cell was PEG‐Ca^2+^‐transfected with pDsRed and observed with a fluorescent microscope. (e), Rice egg cell was cotransfected with pGFP‐ER and pDsRed and observed with a fluorescent microscope. Left and right panels in (b‐d) represent fluorescence and bright‐field images, respectively. Left, middle, and right panels in (e) represent images of a GFP‐derived signal, RFP‐derived signal, and bright‐field, respectively. N in (a) and (e) represents egg nucleus. Arrowheads in (b) and (c) indicate nucleoli in egg nucleus. Bars = 20 μm

When rice egg cells transfected with pGFP‐ER were observed with a confocal laser scanning microscope, the endoplasmic reticulum around the nucleus and at the cytoplasm/cell cortex was visible (Figure 2a). Next, pH2B‐GFP, into which DNA encoding histone H2B‐GFP fusion protein was placed under the 35S promoter‐HSP70 intron, was constructed and PEG‐transfected with rice egg cells. A fluorescent signal was detected in the nucleus and a bright fluorescent signal was observed in the nucleoli (Figure 2b), and the intracellular localization pattern of H2B‐GFP in PEG‐Ca^2+^‐transfected egg cells was same as in egg cells isolated from transgenic rice plants expressing H2B‐GFP proteins under the control of ubiquitin promoter (Figure 2c; Abiko et al., 2013). These results indicated that transiently expressed proteins in egg cells through PEG‐Ca^2+^ transfection were correctly translated, folded, and targeted to their destination organelle. In addition, when egg cells were PEG‐Ca^2+^‐transfected with pDsRED, ~33% of transfected cells showed the RFP signal (Figure 2d and Table 1). The efficiency of PEG‐Ca^2+^ transfection of pGFP‐ER and pH2B‐GFP was ~35% and 20%, respectively (Table 1).

We next tested the dual expression of two kinds of fluorescent proteins. Rice egg cells located in a MMG droplet containing pGFP‐ER and pDsRED were PEG‐transfected and fluorescent signals from both fluorescent proteins in cells were observed. Approximately 43% of transfected egg cells showed fluorescent signals. Notably, of 16 fluorescent‐positive cells, 15 showed signals from both SP‐GFP‐HDEL and DsRED (Figure 2e and Table 2). These suggest that two kinds of plasmid DNAs were delivered into the same egg cell via PEG‐Ca^2+^ transfection and functioned in the cell. This tendency, dual expression in one egg cell, would be highly useful to analyze functions of genes of interest in the cells. When egg cells were transfected with two kinds of plasmids, one is harboring the target gene and the other is plasmid DNA of a fluorescent marker, such as pGFP‐ER, pH2B‐GFP, and pRFP, it can be expected that cells showing a fluorescent signal express the gene(s) of interest and the effect of ectopic and/or overexpression of the target gene in egg cells can be monitored in such fluorescent cells.

**Table 2:** Efficiency of PEG‐Ca^2+^ transfection of rice egg cells with dual plasmids

Plasmid	No. of transfected egg cells	Fluorescent signal				Efficiency (%)
		GFP + RFP	GFP only	RFP only	None	
pGFP‐ER + pDsRed	37	15	1	0	21	43.2%

### 2.3 PEG‐Ca^2+^ transfection of isolated zygotes and subsequent culture of the zygotes

**Figure 3:**
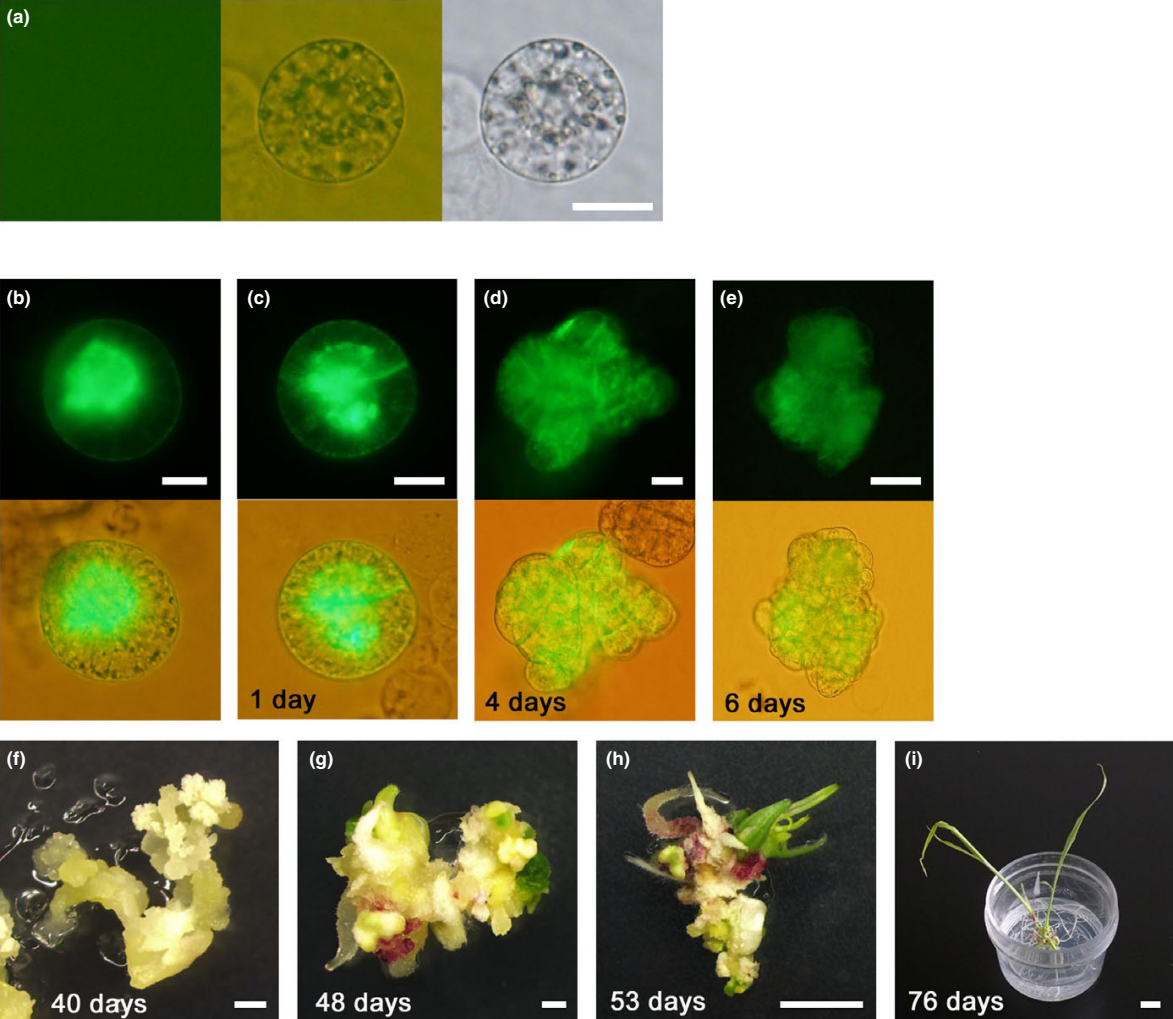
PEG‐Ca^2+^ transfection of rice (a) and maize (b) zygotes and developmental profile of PEG‐Ca^2+^‐transfected maize zygotes (c–i). (a), Rice zygote was PEG‐Ca^2+^‐transfected with pGFP‐ER and observed with fluorescent microscope. (b), Maize zygote was PEG‐Ca^2+^‐transfected with pGFP‐ER and observed with a fluorescent microscope. (c), Two‐celled maize embryo at 1 day after PEG‐Ca^2+^ transfection. (d), Globular embryo‐like structure at 4 days after PEG‐Ca^2+^ transfection. (e), Cell mass at 6 days after PEG‐Ca^2+^ transfection. (f), White callus at 40 days after PEG‐Ca^2+^ transfection. (g), White callus with green spots at 8 days after transferring into regeneration medium (48 days after PEG‐Ca^2+^ transfection). (h), Regenerated shoots at 13 days after transferring to regeneration medium (53 days after PEG‐Ca^2+^ transfection). (i), Plantlet grown from regenerated shoots (76 days after PEG‐Ca^2+^ transfection). Left, middle, and right panels in (a) represent a fluorescent image, merged image, and bright‐field image, respectively. Top and bottom panels in (b–e) represent fluorescent and merged bright‐field and fluorescent images, respectively. Bars = 20 μm in (a–d), 50 μm in (e), 1 mm in (f and g), 5 mm in (h), and 1 cm in (i)

Although rice zygotes were mechanically isolated from pollinated rice flowers by dissection of pollinated ovaries as described by Abiko et al. (2013) and subjected to PEG‐Ca^2+^ transfection with pGFP‐ER, no fluorescent signal was detected in the zygotes (Figure 3a and Table 1). Maize zygotes were isolated by treating dissected ovaries with an enzyme mixture of cellulase, macerozyme, and pectolyase as described in Materials and Methods, and subjected to PEG‐Ca^2+^ transfection. In contrast with rice zygotes, PEG‐Ca^2+^ transfection of the maize zygotes with pGFP‐ER resulted in expression of SP‐GFP‐HDEL with high efficiency (Figure 3b and Table 1).

The failure of transient expression in rice zygotes could be explained by the existence of cell walls around the plasma membranes (Kranz, von Wiegen, & Lörz, 1995; Toda, Ohnishi, & Okamoto, 2016; Uchiumi, Uemura, & Okamoto, 2007), as cell walls impede the access of macromolecules to plasma membrane (Antonelli & Stadler, 1989; Armstrong et al., 1990; Crossway et al., 1986; Davey et al., 1980; Krens et al., 1982; Lurquin, 1979; Shillito et al., 1985). In contrast with rice zygotes, maize zygotes were isolated after treatment with cell wall‐degrading enzymes to loosen cell–cell attachments in female gametophyte tissues, and the cell walls of zygotes were partly or mostly degraded during this procedure. Therefore, upon PEG‐Ca^2+^ transfection of maize zygotes, plasmid DNA could access to plasma membrane of zygotes and be delivered into the cells across the plasma membrane, resulting in expression of fluorescent proteins from the delivered plasmid DNA. Transfection efficiency of maize zygotes with pGFP‐ER was much higher than that of rice egg cells (Table 1). This difference may be explained by the high metabolic activity in zygotes after fertilization.

The maize zygotes showing a fluorescent signal were further cultured, and their developmental profiles and fluorescent signals were observed. The signal from SP‐GFP‐HDEL was detectable in a two‐celled embryo (Figure 3c) and in early embryos consisting of ~10–100 cells (Figure 3d,e) until 6 days after transfection. The cell masses further developed into white calli (Figure 3f). Of 15 zygotes showing the transient SP‐GFP‐HDEL signal, 12 zygotes normally developed into white calli (Table 3), suggesting high developmental efficiency. However, no GFP‐derived signal was detected in the calli. The white callus further regenerated shoots (Figure 3g,h) and formed plantlets (Figure 3i). Although the regeneration efficiency was low (Table 3), the present study provided the possibility that PEG‐transfected maize zygotes can develop into mature plants. This low regeneration rate may be due to the trait of B73 inbred line of maize, whose regeneration efficiency is lower than that in other inbred lines such as A188 and H99 (Hodges, Kamo, Imbrie, & Becwar, 1986; Krakowsky et al., 2006). To check whether possible integration of plasmid DNA into genome DNA and subsequent silencing of the integrated DNA occur, genomic PCR was conducted for the DNA sequence of the SP‐GFP‐HDEL plasmid although GFP‐derived signal was not observed in the regenerated plant. The result indicated that no DNA integration occurs in the regenerated plant during zygote culture (Fig. S1). However, transformed plants will be produced from PEG‐Ca^2+^‐transfected zygotes when increased numbers of zygotes were PEG‐Ca^2+^‐transfected with plasmid DNA and cultured, as the production of stable transformants by PEG‐Ca^2+^ transfection of somatic protoplasts with exogenously applied DNA has been reported in several plants including rice and maize (Armstrong et al., 1990; Datta, Datta, Soltanifar, Donn, & Potrykus, 1992; Lee et al., 1991; Zhang et al., 1991).

**Table 3:** Developmental profile of PEG‐Ca^2+^‐transfected maize zygotes

No. of transfected zygotes	No. of zygotes developed into each growth stage				
	Two‐celled embryo	Globular embryo‐like	Cell mass	White Callus	Plantlet
15	14	14	13	12	1

The present results from PEG‐Ca^2+^ transfection with maize zygotes indicated that transient expression from delivered plasmid DNA in maize zygotes continues for ~6 days at the stage of cell mass and that PEG‐Ca^2+^ treatment of zygotes has little inhibitory effect on the developmental profile of maize zygotes.

## 3 CONCLUSION

To date, procedures for the isolation of female gametes and/or zygotes from flowers have been reported for a wide range of plant species including maize, rice, wheat, barley, tobacco, and Arabidopsis (reviewed in Kranz, 1999 and in Okamoto, 2011). It is likely that the present PEG‐Ca^2+^ transfection system could be applied to most female gametes and zygotes isolated from angiosperms because the cell walls of these isolated egg cells and zygotes are undeveloped or removed during the isolation procedures. The present PEG‐Ca^2+^ transfection system made it possible to deliver plasmid DNA into rice egg cells with 20%–40% efficiency and into maize zygotes with >70% efficiency. Using this system, molecular approaches for gene functions, such as ectopic expression, overexpression, antisense effect, and RNA interference, can be conducted with the direct use of egg cells and zygotes. For such analyses, genes that were specifically or preferentially expressed in female gametes, zygotes, or early embryos (Abiko et al., 2013; Anderson et al., 2013; Ning et al., 2006; Ohnishi et al., 2011; Sprunck et al., 2005; Steffen et al., 2007; Wang et al., 2010; Wuest et al., 2010; Yang et al., 2006) will be noted, as they will function in reproductive or developmental processes, such as gamete differentiation, gamete fusion, and early zygotic development.

In addition, transient expression induced by PEG‐Ca^2+^ transfection within zygotes would be highly suitable for gene editing applications, as transient expression of CRISPR/Cas9 and guide RNA via plasmid DNA in zygotes/early embryos will provide the opportunity for gene editing during early zygotic embryogenesis, and the PEG‐Ca^2+^‐transfected zygotes can develop normally into mature plants.

## 4 EXPERIMENTAL PROCEDURES

### 4.1 Plant materials, isolation of gametes and zygotes

*Oryza sativa* L. cv. Nipponbare plants and transformed rice plants expressing the histone H2B‐GFP fusion protein (Abiko et al., 2013) were grown in an environmental chamber (K30‐7248; Koito Industries, Yokohama, Japan) at 26°C under a 13‐hr light/11‐hr dark photoperiod. Rice egg cells were isolated from flowers of these plants as described by Uchiumi et al. (2006), except for using mannitol solution adjusted to 450 mOsmol/kg H_2_O. *Oryza sativa* L. cv. Yukihikari plants were grown in an environmental chamber (Nippon Medical & Chemical Instruments, Osaka, Japan) at 26°C/24°C under a 12‐hr light/12‐hr dark photoperiod. Rice zygotes were isolated from flowers 4–6 hr after flowering as previously described by Abiko et al. (2013).

The inbred maize (*Zea mays*) lines A188 and B73 were grown in a glasshouse of Japan Tobacco Inc. (Iwata, Japan). Egg cells were isolated from ears (cv A188) as described previously (Kranz et al., 1991). For zygote isolation, maize ears (cv B73) were harvested and pollinated as previously described (Kranz & Brown, 1992; Kranz & Lörz, 1990). After 22–24 hr of pollination, ovule pieces were dissected and zygote isolation was performed according to Kranz et al. (1991) except for the use of enzyme solution of 1% cellulase (Worthington Biochemical Co., NJ), 0.3% macerozyme R10 (Yakult, Tokyo, Japan), and 0.05% pectolyase (Wako, Osaka, Japan).

### 4.2 Vector construction

A signal peptide (SP) of *Vigna mung* cysteine endopeptidase, termed SH‐EP (accession no. X51900), was amplified by PCR using TCTAGATGAAGAAGCTCTTGTGGGT and CCATGGCTTGGCCACTCCAAGAAC as primers and SH‐EP cDNA (Akasofu, Yamauchi, & Minamikawa, 1990) as a template. After subcloning the amplified PCR fragment to pGEM‐T Easy vector (Promega, Madison, WI), the vector was cut by XbaI and NcoI and the excised insert was subcloned into the XbaI‐NcoI site of the 35S promoter‐GFP(S65T) plasmid (Chiu et al., 1996) to produce a pSP‐GFP encoding 26‐amino acid signal peptide followed by GFP. Next, a His‐Asp‐Glu‐Leu (HDEL)‐tail was introduced at the carboxy terminus of SP‐GFP to localize the GFP proteins in the ER. Two oligonucleotides (GATGACTCTCATGATGAGCTGTAGAGCTCGC and GGCCGCGAGCTCTACAGCTCATCATGAGAGT) were annealed, and then the annealed oligo was subcloned into the Bsp1407I–NotI site of pSP‐GFP to produce pSP‐GFP‐HDEL. Using the pSP‐GFP‐HDEL as a template, PCR was further conducted with primers (GGATCCATGAAGAAGCTCTTGTGGGT and GAATTCAGCTCTACAGCTCATCATGAGAGT). After subcloning the amplified PCR fragment into pGEM‐T Easy vector, the sequence was verified. The produced vector was further cut with *Bam*HI and *Eco*RI, and the insert was subcloned to the BamHI‐EcoRI site of pmon30049 (Pang et al., 1996). The constructed plasmid vector, termed pGFP‐ER, was used for the expression of SP‐GFP‐HDEL in egg cells and zygotes.

DNA fragment encoding GFP proteins tagged with histone H2B (H2B‐GFP) were amplified by PCR using primers (GGCCTCTAGAGGATCATGGCGAAGGCAGATAAGAA and GAACGATCGGGAATTTTACTTGTACAGCTCGTCCA) and Ubi promoter::H2B‐GFP vector (Abiko et al., 2013) as a template. Using the amplified PCR fragment and a BamHI‐EcoRI‐digested pGFP‐ER vector, pH2B‐GFP was constructed using an In‐Fusion HD Cloning Kit (Takara Bio, Shiga, Japan) according to the manufacturer's instructions. After verification of the DNA sequence, the plasmid vector, pH2B‐GFP, was used for the expression of H2B‐GFP.

The 2.0‐kb PmeI‐HpaI fragment, which carried the promoter and the first intron of the ubiquitin gene of maize (Christensen, Sharrock, & Quailet, 1992) from pSB200 (Komori et al., 2004), the 1.7‐kb EcoRV fragment carrying the ccdB cassette for the Gateway system (Invitrogen, Carlsbad, CA) and the 0.3‐kb HpaI‐SgfI fragment possessing the 3′ signal of Nos gene (Depicker, Stachel, Dhaese, Zambryski, & Goodman, 1982) were cloned into the SmaI site of pLC41 (GenBank accession no. LC215698). The ccdB cassette was replaced with the 0.8‐kb DsRed2 gene from pDsRed2 (Takara Bio) using the Gateway reaction. The 0.2‐kb fragment, which carried the 3′ signal of cauliflower mosaic virus 35S transcript (Odell, Nagy, & Chua, 1985), was amplified by PCR using primers (CAACACTTACGCGATCGCTGAAATCACCAGTCTCTCTCTACAAA and GCTCGGATACCGCGATCGCACTGGATTTTGGTTAGG) and inserted to the AsiSI site behind the Nos 3′ fragment of the resultant plasmid as an additional 3′ signal. Then, the DsRed2 gene cassette was cloned into the pCR‐Blunt II‐TOPO vector using the Zero Blunt TOPO PCR cloning system (Invitrogen). The produced vector, termed pDsRed, was used for the expression of RFP proteins in gametes.

### 4.3 PEG‐Ca^2+^ transfection of egg cells and zygotes with plasmid DNA

PEG‐Ca^2+^ transfection using gametes and zygotes was conducted as described by Yoo et al. (2007) and Zhai et al. (2009) with major modifications. Four to eight rice egg cells or zygotes isolated from rice flowers were transferred into a 0.5–1.0 μl droplet of mannitol solution (450 mOsmol/kg H_2_O) overlaid with mineral oil on a coverslip. The cells were washed twice by transferring the cells into droplets of MMG solution (4 mM MES‐KOH, pH 5.7, 15 mM MgCl_2_ in mannitol solution of 450 mOsmol/kg H_2_O) on coverslips. The washed cells were transferred into a 1.0 μl droplet of MMG solution containing plasmid DNA (MMG‐DNA), and then 1.0 μl of PEG solution (30% PEG4000 (Sigma‐Aldrich, St. Louis, MO) and 100 mM CaCl_2_ in mannitol solution in 450 mOsmol/kg H_2_O) was merged into the MMG‐DNA droplet where the egg cells were held. Immediately after the droplet merge, the solution in the droplet was gently mixed with a glass capillary for 1 min. After incubation for 5 min, approximately half of the volume of the solution in the mixed droplet was sucked out using a glass capillary connected with manual injector. Next, N6Z culture medium (Uchiumi et al., 2007) or mannitol solution was delivered into a droplet, and after gentle mixing of the droplet, approximately half of the volume of the solution was removed from the droplet. This washing step was repeated four to five times. Then, cells were further washed by transferring the cells into fresh droplets of N6Z medium or mannitol solution three times. Thereafter, as described by Nakajima, Uchiumi, and Okamoto (2010), the egg cells were cultured in N6Z droplets, in which two to three cell aggregates of rice suspension‐cultured cells were co‐cultured. The rice suspension‐cultured cells (Line Oc) was provided by Riken Bio‐Resource Center, Tsukuba, Japan. The PEG‐Ca^2+^‐transfected zygotes were cultured in a Millicell‐CM insert (Merck Millipore, Darmstadt, Germany) with N6Z medium as described (Uchiumi et al., 2007). Egg cells and zygotes were cultured at 25°C in darkness.

Maize egg cells and zygotes were subjected to PEG‐Ca^2+^ transfection using the same procedures as for rice egg cells, except for the following. Instead of mannitol solution of 450 mOsmol/kg H_2_O, a mannitol solution of 650 mOsmol/kg H_2_O was used in the MMG and PEG solutions. In addition, washing maize zygotes with MMG was conducted on 1.5% solidified Gelrite to prevent the attachment of zygotes to the coverslip, and then, the washed zygotes were PEG‐Ca^2+^‐transfected in the same way as the rice egg cells. After PEG‐Ca^2+^ transfection, maize egg cells or zygotes were cultured in a Millicell‐CM insert (Merck Millipore) with MSO medium as described (Kranz & Lörz, 1993). Egg cells and zygotes were cultured at 25°C in darkness.

### 4.4 Microscopic observations

Cells transfected with pGFP‐ER or pH2B‐GFP were observed under a BX‐71 inverted fluorescence microscope (Olympus, Tokyo, Japan) with 460‐ to 490‐nm excitation and 510‐ to 550‐nm emission wavelengths (U‐MWIBA2 mirror unit; Olympus). The fluorescence of DsRed proteins in cells was observed with 520‐ to 550‐nm excitation and >580‐nm emission wavelengths (U‐MWIG mirror unit; Olympus). Digital images of gametes, zygotes, and their resulting embryos were obtained using a cooled charge‐coupled device camera (Penguin 600CL; Pixera, Los Gatos, CA, USA) and InStudio software (Pixera). In addition to observation with fluorescence microscope, rice egg cells, which were PEG‐transfected with pGFP‐ER, were observed with an LSM 710 CLS microscope (Carl Zeiss, Jena, Germany) with 488‐nm excitation and 505‐ to 530‐nm emission wavelengths.

## AUTHOR CONTRIBUTIONS

K.N. and E.T. conducted PEG transfection and plasmid construction; M.T. performed plasmid construction for a plasmid construct for RFP expression; K.N. provided suggestions and technical assistances; T.O. supervised the experiments and wrote the article.

## References

[pld310-bib-0001] Abiko, M. , Maeda, H. , Tamura, K. , Hara‐Nishimura, I. , & Okamoto, T. (2013). Gene expression profiles in rice gametes and zygotes: Identification of gamete‐enriched genes and up‐ or down‐regulated genes in zygotes after fertilization. Journal of Experimental Botany, 64a, 1927–1940. 10.1093/jxb/ert054 PMC3638821 23570690

[pld310-bib-0002] Akasofu, H. , Yamauchi, D. , & Minamikawa, T. (1990). Nucleotide sequence of the gene for the *Vigna mungo* sulfhydryl‐endopeptidase (SH‐EP). Nucleic Acid Research, 18, 1892. 10.1093/nar/18.7.1892 PMC330615 2336365

[pld310-bib-0003] An, C. I. , Sawada, A. , Kawaguchi, Y. , Fukusaki, E. , & Kobayashi, A. (2005). Transient RNAi induction against endogenous genes in Arabidopsis protoplasts using in vitro‐prepared double‐stranded RNA. Bioscience, Biotechnology, and Biochemistry, 69, 415–418. 10.1271/bbb.69.415 15725671

[pld310-bib-0004] Anderson, S. N. , Johnson, C. S. , Jones, D. S. , Conrad, L. J. , Gou, X. , Russell, S. D. , & Sundaresan, V. (2013). Transcriptomes of isolated Oryza sativa gametes characterized by deep sequencing: Evidence for distinct sex‐dependent chromatin and epigenetic states before fertilization. Plant Journal, 76, 729–741. 24215296 10.1111/tpj.12336

[pld310-bib-0005] Antonelli, N. M. , & Stadler, J. (1989). Chemical methods for efficient direct gene transfer to maize cells: Treatment with polyethylene glycol or Polybrene. Journal of Genetics and Plant Breeding, 43, 113–122.

[pld310-bib-0006] Armstrong, C. L. , Petersen, W. L. , Buchholz, W. G. , Bowen, B. A. , & Sulc, S. L. (1990). Factors affecting PEG‐mediated stable transformation of maize protoplasts. Plant Cell Reports, 9, 335–339. 24226946 10.1007/BF00232864

[pld310-bib-0007] Belhaj, K. , Chaparro‐Garcia, A. , Kamoun, S. , & Nekrasov, V. (2013). Plant genome editing made easy: Targeted mutagenesis in model and crop plants using the CRISPR/Cas system. Plant Methods, 9, 39. 24112467 10.1186/1746-4811-9-39 PMC3852272

[pld310-bib-0008] Chiu, L. W. , Niwa, Y. , Zeng, W. , Hirano, T. , Kobayashi, H. , & Sheen, J. (1996). Engineered GFP as a vital reporter in plants. Current Biology, 6, 325–330. 8805250 10.1016/s0960-9822(02)00483-9

[pld310-bib-0009] Christensen, A. H. , Sharrock, R. A. , & Quailet, P. H. (1992). Maize polyubiquitin genes: Structure, thermal perturbation of expression and transcript splicing, and promoter activity following transfer to protoplasts by electroporation. Plant Molecular Biology, 18, 675–689. 1313711 10.1007/BF00020010

[pld310-bib-0010] Crossway, A. , Hauptli, H. , Houck, C. M. , Irvine, J. M. , Oakes, J. V. , & Perani, L. A. (1986). Micromanipulation techniques in plant biotechnology. BioTechniques, 4, 320–334.

[pld310-bib-0011] Datta, S. K. , Datta, K. , Soltanifar, N. , Donn, G. , & Potrykus, I. (1992). Herbicide‐resistant Indica rice plants from IRRI breeding line IR72 after PEG‐mediated transformation of protoplasts. Plant Molecular Biology, 20, 619–629. 1333295 10.1007/BF00046447

[pld310-bib-0012] Davey, M. R. , Cocking, E. C. , Freeman, J. , Pearce, N. , & Tudor, I. (1980). Transformation of petunia protoplasts by isolated agrobacterium plasmids. Plant Science Letters, 18, 307–313.

[pld310-bib-0013] Davey, M. R. , Rech, E. L. , & Mulligan, B. J. (1989). Direct DNA transfer to plant cells. Plant Molecular Biology, 13, 273–285. 2491654 10.1007/BF00025315

[pld310-bib-0014] Depicker, A. , Stachel, S. , Dhaese, P. , Zambryski, P. , & Goodman, H. M. (1982). Nopaline synthase: Transcript mapping and DNA sequence. Journal of Molecular and Applied Genetics, 1, 561–573. 7153689

[pld310-bib-0015] Guignard, M. L. (1899). Sur les antherozoides et la double copulation sexuelle chez les vegetaux angiosperms. Rev. Gén. Bot., 11, 129–135. 10.1016/s0764-4469(01)01346-4 11460830

[pld310-bib-0016] Haseloff, J. , Siemering, K. R. , Prasher, D. C. , & Hodge, S. (1997). Removal of a cryptic intron and subcellular localization of green fluorescent protein are required to mark transgenic Arabidopsis plants brightly. Proceedings of the National Academy of Sciences of the United States of America, 94, 2122–2127. 9122158 10.1073/pnas.94.6.2122 PMC20051

[pld310-bib-0017] Hayashi, Y. , Yamada, K. , Shimada, T. , Matsushima, R. , Nishizawa, N. , Nishimura, M. , & Hara‐Nishimura, I. (2001). A proteinase‐storing body that prepares for cell death or stresses in the epidermal cells of Arabidopsis. Plant and Cell Physiology, 42, 894–899. 11577182 10.1093/pcp/pce144

[pld310-bib-0018] Hodges, T. K. , Kamo, K. K. , Imbrie, C. W. , & Becwar, M. R. (1986). Genotype specificity of somatic embryogenesis and regeneration in maize. Biotechnology, 4, 219–223.

[pld310-bib-0019] Jinek, M. , Chylinski, K. , Fonfara, I. , Hauer, M. , Doudna, J. A. , & Charpentier, E. (2012). A programmable dual‐RNA‐guided DNA endonuclease in adaptive bacterial immunity. Science, 337, 816–821. 22745249 10.1126/science.1225829 PMC6286148

[pld310-bib-0020] Komori, T. , Ohta, S. , Murai, N. , Takakura, Y. , Kuraya, Y. , Suzuki, S. , … Nitta, N. (2004). Map‐based cloning of a fertility restorer gene, Rf‐1, in rice (*Oryza sativa* L.). Plant Journal, 37, 315–325. 14731253 10.1046/j.1365-313x.2003.01961.x

[pld310-bib-0021] Krakowsky, M. D. , Lee, M. , Garay, L. , Woodman‐Clikeman, W. , Long, M. J. , Sharopova, N. , … Wang, K. (2006). Quantitative trait loci for callus initiation and totipotency in maize (*Zea mays* L.). TAG. Theoretical and Applied Genetics, 113, 821–830. 16896717 10.1007/s00122-006-0334-y

[pld310-bib-0022] Kranz, E. (1999). In vitro fertilization with isolated single gametes. Methods in Molecular Biology, 111, 259–267. 10080993 10.1385/1-59259-583-9:259

[pld310-bib-0023] Kranz, E. , Bautor, J. , & Lörz, H. (1991). In vitro fertilization of single, isolated gametes of maize mediated by electrofusion. Sexual Plant Reproduction, 4, 12–16.

[pld310-bib-0024] Kranz, E. , & Brown, P. T. H. (1992). Micromanipulation and in vitro fertilization with single pollen grains and isolated gametes of maize In Cresti M., & Tiezzi A. (Eds.), Sexual plant reproduction (pp. 173–183). Berlin, Heidelberg, New York: Springer.

[pld310-bib-0025] Kranz, E. , & Lörz, H. (1990). Micromanipulation and in vitro fertilization with single pollen grains of maize. Sexual Plant Reproduction, 3, 160–169.

[pld310-bib-0026] Kranz, E. , & Lörz, H. (1993). In vitro fertilization with isolated, single gametes results in zygotic embryogenesis and fertile maize plants. Plant Cell, 5, 739–746. 12271084 10.1105/tpc.5.7.739 PMC160312

[pld310-bib-0027] Kranz, E. , von Wiegen, P. , & Lörz, H. (1995). Early cytological events after induction of cell division in egg cells and zygote development following *in vitro* fertilization with angiosperm gametes. Plant Journal, 8, 9–23.

[pld310-bib-0028] Krens, F. A. , Molendijk, L. , Wullems, G. J. , & Schilperoort, R. A. (1982). In vitro transformation of plant protoplasts with Ti‐plasmid DNA. Nature, 296, 72–74.

[pld310-bib-0029] Lee, N. , Wang, Y. , Yang, J. , Ge, K. , Huang, S. , Tan, J. , & Testa, D. (1991). Efficient transformation and regeneration of rice small cell groups. Proceedings of the National Academy of Sciences of the United States of America, 88, 6389–6393. 11607199 10.1073/pnas.88.15.6389 PMC52090

[pld310-bib-0030] Lurquin, P. F. (1979). Entrapment of plasmid DNA by liposomes and their interactions with plant protoplasts. Nucleic Acids Research, 6, 3773–3784. 493123 10.1093/nar/6.12.3773 PMC327977

[pld310-bib-0031] Mikami, M. , Toki, S. , & Endo, M. (2015). Comparison of CRISPR/Cas9 expression constructs for efficient targeted mutagenesis in rice. Plant Molecular Biology, 88, 561–572. 26188471 10.1007/s11103-015-0342-x PMC4523696

[pld310-bib-0032] Munro, S. , & Pelham, H. B. R. (1987). A C‐terminal signal prevents secretion of luminal ER proteins. Cell, 48, 899–907. 3545499 10.1016/0092-8674(87)90086-9

[pld310-bib-0033] Nakajima, K. , Uchiumi, T. , & Okamoto, T. (2010). Positional relationship between the gamete fusion site and the first division plane in the rice zygote. Journal of Experimental Botany, 61, 3101–3105. 20462944 10.1093/jxb/erq131 PMC2892148

[pld310-bib-0034] Nawaschin, S. (1898). Revision der Befruchtungsvorgange bei Lilium martagon und Fritillaria tenella. Bulletin de l'Académie Impériale des Sciences de Saint‐Pétersbourg, 9, 377–382.

[pld310-bib-0035] Ning, J. , Peng, X.‐B. , Qu, L.‐H. , Xin, H. P. , Yan, T. T. , & Sun, M. X. (2006). Differential gene expression in egg cells and zygotes suggests that the transcriptome is restructed before the first zygotic division in tobacco. FEBS Letters, 580, 1747–1752. 16510144 10.1016/j.febslet.2006.02.028

[pld310-bib-0036] Odell, J. T. , Nagy, F. , & Chua, N. H. (1985). Identification of DNA sequences required for activity of the cauliflower mosaic virus 35S promoter. Nature, 313, 810–812. 3974711 10.1038/313810a0

[pld310-bib-0037] Ohnishi, T. , Takanashi, H. , Mogi, M. , Takahashi, H. , Kikuchi, S. , Yano, K. , … Tsutsumi, N. (2011). Distinct gene expression profiles in egg and synergid cells of rice as revealed by cell type‐specific microarrays. Plant Physiology, 155, 881–891. 21106719 10.1104/pp.110.167502 PMC3032473

[pld310-bib-0038] Okamoto, T. (2011). In vitro fertilization with isolated rice gametes: Production of zygotes and zygote and embryo culture. Methods in Molecular Biology, 710, 17–27. 21207258 10.1007/978-1-61737-988-8_2

[pld310-bib-0039] Pang, S. Z. , DeBoer, D. L. , Wan, Y. , Ye, G. , Layton, J. G. , Neher, M. K. , … Fromm, M. E. (1996). An improved green fluorescent protein gene as a vital marker in plants. Plant Physiology, 112, 893–900. 8938400 10.1104/pp.112.3.893 PMC158016

[pld310-bib-0040] Pelham, H. B. R. (1989). Control of protein exit from the endoplasmic reticulum. Annual Review of Cell Biology, 5, 1–23. 10.1146/annurev.cb.05.110189.000245 2688704

[pld310-bib-0041] Raghavan, V. (2003). Some reflections on double fertilization, from its discovery to the present. New Phytologist, 159, 565–583. 10.1046/j.1469-8137.2003.00846.x 33873607

[pld310-bib-0042] Ridge, R. W. , Uozumi, Y. , Plazinski, J. , Hurley, U. A. , & Williamson, R. E. (1999). Developmental transitions and dynamics of the cortical ER of Arabidopsis cells seen with green fluorescent protein. Plant and Cell Physiology, 40, 1253–1261. 10682347 10.1093/oxfordjournals.pcp.a029513

[pld310-bib-0043] Russell, S. D. (1992). Double fertilization. International Review of Cytology, 40, 357–390.

[pld310-bib-0044] Sheen, J. (2001). Signal transduction in maize and Arabidopsis mesophyll protoplasts. Plant Physiology, 127, 1466–1475. 11743090 PMC1540179

[pld310-bib-0045] Shillito, R. D. , Saul, M. W. , Paszkowski, J. , Muller, M. , & Potrykus, I. (1985). High efficiency direct gene transfer to plants. Bio/Technology, 3, 1099–1103.

[pld310-bib-0046] Sprunck, S. , Baumann, U. , Edwards, K. , Langridge, P. , & Dresselhaus, T. (2005). The transcript composition of egg cells changes significantly following fertilization in wheat (*Triticum aestivum* L.). Plant Journal, 41, 660–672. 15703054 10.1111/j.1365-313X.2005.02332.x

[pld310-bib-0047] Sprunck, S. , Rademacher, S. , Vogler, F. , Gheyselinck, J. , Grossniklaus, U. , & Dresselhaus, T. (2012). Egg cell–secreted EC1 triggers sperm cell activation during double fertilization. Science, 338, 1093–1097. 23180860 10.1126/science.1223944

[pld310-bib-0048] Steffen, J. G. , Kang, I. H. , Macfarlane, J. , & Drews, G. N. (2007). Identification of genes expressed in the Arabidopsis female gametophyte. Plant Journal, 51, 281–292. 17559508 10.1111/j.1365-313X.2007.03137.x

[pld310-bib-0049] Toda, E. , Ohnishi, Y. , & Okamoto, T. (2016). Development of polyspermic rice zygotes. Plant Physiology, 171, 206–214. 26945052 10.1104/pp.15.01953 PMC4854695

[pld310-bib-0050] Uchiumi, T. , Komatsu, S. , Koshiba, T. , & Okamoto, T. (2006). Isolation of gametes and central cells from *Oryza sativa* L. Sexual Plant Reproduction, 19, 37–45.

[pld310-bib-0051] Uchiumi, T. , Uemura, I. , & Okamoto, T. (2007). Establishment of an in vitro fertilization system in rice (*Oryza sativa* L.). Planta, 226, 581–589. 17361458 10.1007/s00425-007-0506-2

[pld310-bib-0052] Wang, Y. Y. , Kuang, A. , Russell, S. D. , & Tian, H. Q. (2006). In vitro fertilization as a tool for investigating sexual reproduction of angiosperm. Sexual Plant Reproduction, 19, 103–115.

[pld310-bib-0053] Wang, D. , Zhang, C. Q. , Hearn, D. J. , Kang, I. H. , Punwani, J. A. , Skaggs, M. I. , … Yadegari, R. (2010). Identification of transcription‐factor genes expressed in the Arabidopsis female gametophyte. BMC Plant Biology, 10, 110. 20550711 10.1186/1471-2229-10-110 PMC3236301

[pld310-bib-0054] Wuest, S. E. , Vijverberg, K. , Schmidt, A. , Weiss, M. , Gheyselinck, J. , Lohr, M. , … Grossniklaus, U. (2010). Arabidopsis female gametophyte gene expression map reveals similarities between plant and animal gametes. Current Biology, 20, 506–512. 20226671 10.1016/j.cub.2010.01.051

[pld310-bib-0055] Yang, H. , Kaur, N. , Kiriakopolos, S. , & McCormick, S. (2006). EST generation and analyses towards identifying female gametophyte‐specific genes in *Zea mays* L. Planta, 224, 1004–1014. 16718485 10.1007/s00425-006-0283-3

[pld310-bib-0056] Yoo, S. D. , Cho, Y. H. , & Sheen, J. (2007). Arabidopsis mesophyll protoplasts: A versatile cell system for transient gene expression analysis. Nature Protocols, 2, 1565–1572. 17585298 10.1038/nprot.2007.199

[pld310-bib-0057] Zhai, Z. , Sooksa‐nguan, T. , & Vatamaniuk, O. K. (2009). Establishing RNA interference as a reverse‐genetic approach for gene functional analysis in protoplasts. Plant Physiology, 149, 642–652. 19005083 10.1104/pp.108.130260 PMC2633838

[pld310-bib-0058] Zhang, W. , McElroy, D. , & Wu, R. (1991). Analysis of rice ACT1 5 region activity in transgenic rice plants. Plant Cell, 3, 1155–1165. 1821763 10.1105/tpc.3.11.1155 PMC160082

