# Supplementary material for: Development of gene expression system in egg cells and zygotes isolated from rice and maize
Source: Plant Direct. 2017 Sep 6;1(3):e00010. doi: 10.1002/pld3.10 (PMC6508540; doi:10.1002/pld3.10)
Supplement: Supplementary file 1 [file PLD3-1-e00010-s001.pdf]

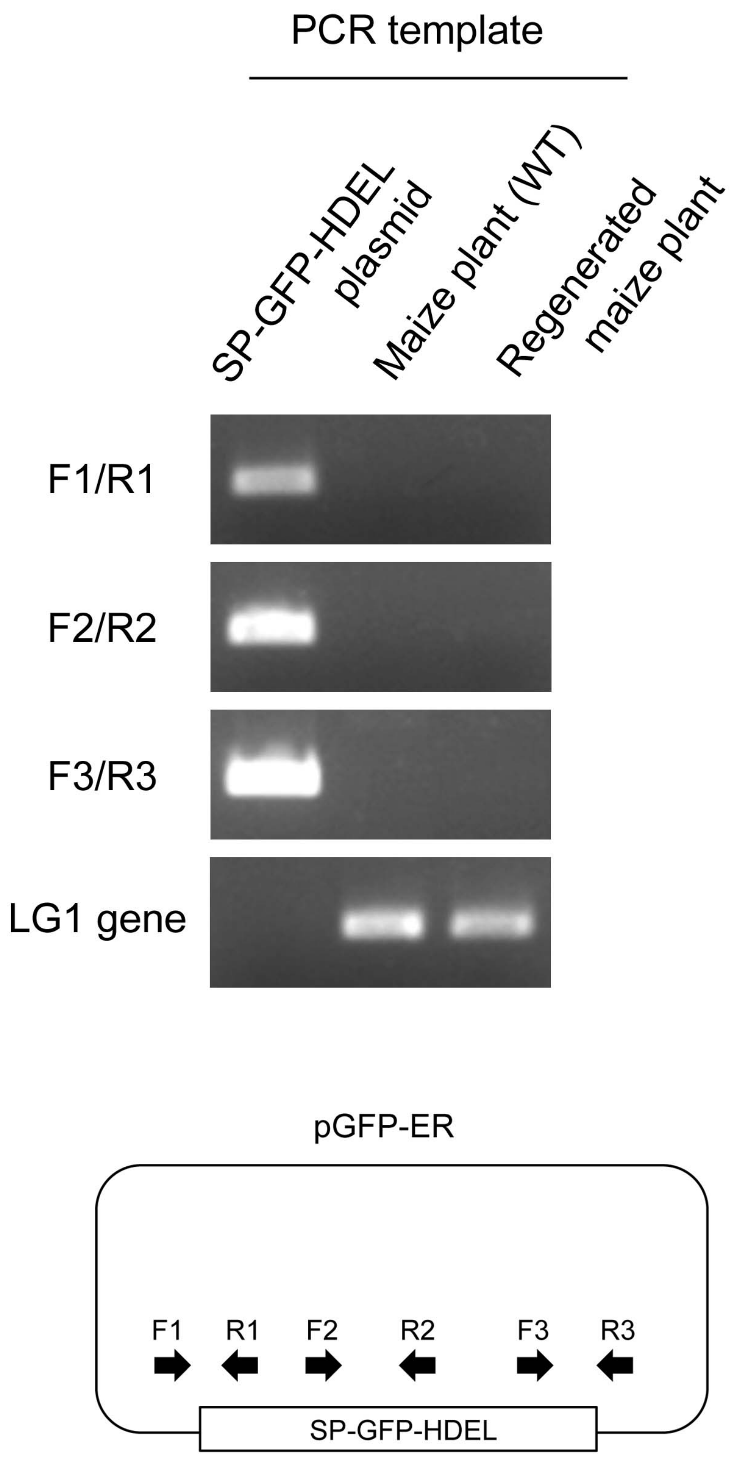

Figure S1. Genome DNAs were isolated from wild type maize plant and the maize plant regenerated from PEG-transfected zygote with pGFP-ER plasmid DNA. PCR was performed on isolated genome DNAs and pGFP-ER using primer sets for the plasmid DNA (F1 and R1, ATTGTAATGCAGATACCAAGCG and GTGGTGCAGATGAACTTCAGG; F2 and R2, TCGTGACCACCTTCACCTACG and CTTGATGCCGTTCTTCTGCTTG; F3 and R3, ACAACCACTACCTGAGCACCCAG and CATGATTACGCCAAGCTGCG) and for *LIGULELESS1* (*LG1*, GRMZM2G036297) gene (TCAGTAGGCCACTCGGTCAT and ACTCGTGAGATCGAGCAACA).
