# Supplementary material for: Development of gene expression system in egg cells and zygotes isolated from rice and maize
Source: Plant Direct. 2017 Sep 6;1(3):e00010. doi: 10.1002/pld3.10 (PMC6508540; doi:10.1002/pld3.10)
Supplement: Supplementary file 2 [file PLD3-1-e00010-s002.pdf]

Table S1. Effect of PEG concentration on the expression of proteins from delivered plasmid DNA

PEG concentration (%)	No. of infected cells	Fluorescent signal		
		++	+	-
20	4	0	0	4
30	4	2	0	2
40	4	0	1	3

Table S2. Effect of plasmid DNA concentration on the expression of proteins from delivered plasmid DNA

plasmid DNA concentration (ng/μl)	No. of infected cells	Fluorescent signal					
		13h after infection			19h after infection		
		++	+	-	++	+	-
17	5	0	2	3	2	0	3
68	8	1	3	4	4	0	4
272	5	2	0	3	2	0	3
